# Early Renal Effects After Radioactive Iodine Therapy in Differentiated Thyroid Cancer: A Prospective Observational Study

**DOI:** 10.3390/diagnostics16193123

**Published:** 2026-09-25

**Authors:** Muhammed Enes Solakoğlu, Adem Maman, Esra Laloğlu

**Affiliations:** 1 Department of Nuclear Medicine, Faculty of Medicine, Atatürk University, 25240 Erzurum, Türkiye; adem.maman@atauni.edu.tr; 2 Department of Medical Biochemistry, Faculty of Medicine, Atatürk University, 25240 Erzurum, Türkiye; esra.laloglu@atauni.edu.tr

**Keywords:** differentiated thyroid cancer, radioactive iodine therapy, iodine-131, urinary biomarkers, kidney injury molecule-1, netrin-1, cystatin C, β2-microglobulin

## Abstract

**Background/Objectives**: Radioactive iodine (RAI) therapy is widely used after thyroidectomy for differentiated thyroid cancer, but early renal tubular changes may not be reflected by conventional renal function parameters. We prospectively evaluated urinary tubular biomarker changes across three administered RAI activities. **Methods:** We included 120 patients, with 40 in each activity group (1.11, 3.70, and 5.55 GBq). Blood was collected at baseline and day 7, and urine at baseline, day 1, and day 7. Urinary kidney injury molecule-1, netrin-1, cystatin C, and β2-microglobulin were measured by enzyme-linked immunosorbent assay; conventional renal parameters were also assessed. **Results:** All four biomarkers increased significantly from baseline to Day 1 in all activity groups. Percentage increases were numerically greater at higher administered activities; however, baseline-adjusted mixed-effects analysis showed no significant activity-group effect or activity group × time interaction. Absolute biomarker levels partially declined by Day 7 and remained numerically above baseline, whereas urinary creatinine-normalized biomarker ratios remained significantly elevated. Serum creatinine decreased and creatinine-based eGFR increased by Day 7, whereas urinary albumin, protein, and phosphate showed no consistent changes across the activity groups. **Conclusions:** The concordant early changes observed in urinary tubular biomarkers, without deterioration in conventional renal function parameters, support an early subclinical tubular response following RAI therapy. Although percentage increases were numerically greater at higher administered activities, baseline-adjusted mixed-effects analyses did not support an independent activity-related effect. Therefore, these findings should not be interpreted as evidence of clinically significant renal injury. Longer-term studies are needed to determine the persistence, reversibility, and clinical significance of these findings.

## 1. Introduction

Radioactive iodine (RAI) therapy is widely used after total thyroidectomy in patients with differentiated thyroid cancer (DTC) for remnant ablation, adjuvant treatment, and the treatment of metastatic disease [1]. The administered activity varies according to the clinical indication and patient risk, resulting in variable levels of radiation exposure to nontarget organs because of the physiological distribution and excretion of I-131 [1,2,3]. Because RAI is eliminated predominantly through the kidneys, renal tissue is exposed to radiation during glomerular filtration and tubular handling [3].

Experimental studies have shown that structural and cellular changes may develop in renal tissue following RAI administration [4,5,6]. In an experimental animal study, Aktoz et al. demonstrated histopathological renal tubular injury after RAI administration and reported findings suggesting that amifostine may reduce these changes [4]. Another experimental study showed that renal cellular proliferation and apoptosis began within the first week after RAI administration and became more pronounced with longer follow-up [5]. In a more recent study in rats, long-term renal histopathological changes were also reported following RAI administration [6]. Although these experimental findings support the renal effects of RAI, clinical evidence regarding its early renal effects in humans remains limited. Available human studies have evaluated microalbuminuria or conventional renal parameters such as serum creatinine, blood urea nitrogen, and eGFR; however, early tubular changes have not been directly investigated [7,8]. In addition, these studies have several limitations with respect to study design and the renal parameters evaluated. To the best of our knowledge, no human study has prospectively evaluated the renal effects of RAI therapy.

Conventional renal function markers may remain unchanged despite early tubular changes and may therefore be insufficient for detecting subclinical changes [9,10,11]. Biomarkers such as kidney injury molecule-1 (KIM-1), netrin-1, cystatin C (CysC), and β2-microglobulin (β2M) have been investigated as early indicators of renal tubular stress or injury [10,11,12,13,14].

We hypothesized that RAI therapy would lead to early increases in urinary tubular biomarkers without a marked deterioration in conventional renal function parameters and that these increases would be more pronounced at higher administered activities. Accordingly, in this prospective observational study, we aimed to evaluate temporal changes in urinary KIM-1, netrin-1, CysC, and β2M levels following RAI therapy, to compare these changes across three different administered activity groups, and to simultaneously examine changes in routine biochemical renal function parameters. The findings are expected to contribute to a better understanding of the early renal responses that may occur following RAI therapy and to future research on biomarker-based renal assessment and the long-term clinical significance of these changes.

## 2. Materials and Methods

### 2.1. Study Design and Patient Selection

This prospective, single-center observational study was conducted at the Department of Nuclear Medicine, Faculty of Medicine, Atatürk University, Erzurum, Turkey, between June 2024 and May 2025. The final analysis included 120 patients with DTC who had undergone total thyroidectomy and received RAI in accordance with the 2015 American Thyroid Association guidelines [15]. Treatment decisions, including selection of the administered RAI activity, were made solely as part of routine clinical care according to clinical indication and patient risk; the study protocol did not influence or modify these decisions. RAI was administered orally at 1.11, 3.70, or 5.55 GBq, with 40 patients included in each activity group. Patients were instructed to follow a low-iodine diet for at least 2 weeks before treatment, and levothyroxine was withdrawn for 3–4 weeks to achieve a serum thyroid-stimulating hormone (TSH) level of ≥30 mIU/L. Thyroid hormone withdrawal was the routine preparation protocol at our center during the study period.

After RAI administration, patients were advised to maintain adequate hydration. Levothyroxine was resumed on day 1 at 1.5–2.0 μg/kg/day, with the dose adjusted for age. Patients were discharged in accordance with national radiation-safety guidelines.

Eligibility criteria were histopathologically confirmed DTC, previous total thyroidectomy, planned RAI therapy, and age 18–75 years. Exclusion criteria were a baseline estimated glomerular filtration rate (eGFR) < 30 mL/min/1.73 m^2^, severe hepatic or cardiac insufficiency, a concurrent malignancy other than DTC, use of nephrotoxic medications, or use of medications known to affect renal injury biomarkers. Patients with incomplete follow-up or missing data were excluded from the final analysis. Enrollment continued until 40 patients with complete follow-up were included in each activity group (Figure 1).

The study was approved by the Non-Interventional Clinical Research Ethics Committee of Atatürk University Faculty of Medicine (Meeting No. 4; Decision No. 22; 7 June 2024) and conducted in accordance with the Declaration of Helsinki. All participants provided written informed consent.

### 2.2. Sample Collection and Storage

Blood and urine samples were collected at baseline before RAI administration and on day 7 after treatment; urine samples alone were collected on day 1, approximately 24 h after RAI administration and before resumption of levothyroxine. Urinary creatinine (uCr), urinary albumin (uAlb), urinary protein (uPro), urinary phosphate (uP), and routine urinalysis parameters, including specific gravity, pH, glucose, and leukocyte esterase, were measured at baseline and on day 7. Urine aliquots collected at baseline, day 1, and day 7 were used for the measurement of KIM-1, netrin-1, CysC, and β2M.

Before centrifugation, radioactivity levels of up to 55.5 MBq (1.5 mCi) were measured in tubes containing day 1 urine samples. In accordance with the as-low-as-reasonably-achievable principle, radiation-protection measures were applied, and the volume processed from day 1 samples was restricted to less than 1 mL for most patients. Urine samples were centrifuged at 2000× *g* for 10 min at 4 °C to remove cellular debris. The resulting supernatants were transferred to 1.5-mL tubes and stored at −80 °C until analysis.

Day 1 aliquots were prioritized for the measurement of KIM-1, netrin-1, CysC, and β2M, leaving insufficient volume for the measurement of uCr, uAlb, uPro, and uP. Stored urine samples underwent a single freeze–thaw cycle before analysis. Laboratory analyses were performed only after at least five physical half-lives of ^131^I had elapsed following collection of the final urine sample.

### 2.3. Biomarker Measurements and Laboratory Analyses

Urinary KIM-1, Netrin-1, CysC, and β2M levels were analyzed concurrently during the same analytical session to minimize interassay analytical variation. Urinary KIM-1 levels were measured using the Human KIM-1 ELISA Kit (Cat. No. E1099Hu; BTLAB, Jiaxing, China), Netrin-1 levels using the Human Netrin-1 ELISA Kit (Cat. No. E1277Hu; BTLAB, Jiaxing, China), CysC levels using the Human CysC ELISA Kit (Cat. No. E1104Hu; BTLAB, Jiaxing, China), and β2M levels using the Human B2M ELISA Kit (Cat. No. EA0039Hu; BTLAB, Jiaxing, China), according to the manufacturers’ instructions. All measured concentrations were within the manufacturers’ specified assay ranges. According to the manufacturers, the intra-assay and inter-assay coefficients of variation were <8% and <10%, respectively.

Urinary biomarker levels were normalized to uCr levels to reduce variability resulting from differences in urine concentration. uCr measurements were performed on a Beckman Coulter AU5800 analyzer (Beckman Coulter, Brea, CA, USA) using the colorimetric modified Jaffe method.

Serum creatinine (sCr), blood urea nitrogen (BUN), aspartate aminotransferase (AST), alanine aminotransferase (ALT), lactate dehydrogenase (LDH), albumin, prealbumin, and total protein were analyzed using a Beckman Coulter AU5800 analyzer. Complete blood count parameters, including leukocyte, neutrophil, lymphocyte, erythrocyte, and platelet counts and hemoglobin levels, were analyzed using a Sysmex XN-9000 analyzer (Sysmex Corporation, Kobe, Japan). TSH, triiodothyronine (T3), and thyroxine (T4) levels were measured using a Beckman Coulter DXI 800 analyzer (Beckman Coulter, Inc., Brea, CA, USA). Routine urinalysis was performed using a DIRUI MUS-3600 analyzer (DIRUI Industrial Co., Ltd., Changchun, China). eGFR was calculated from sCr using the 2021 race-free Chronic Kidney Disease Epidemiology Collaboration creatinine equation and expressed in mL/min/1.73 m^2^.

### 2.4. Statistical Analysis

Statistical analyses were performed using IBM SPSS Statistics for Windows, Version 25.0 (IBM Corp., Armonk, NY, USA). The normality of continuous variables was assessed using histogram plots, the Kolmogorov–Smirnov test, and the Shapiro–Wilk test. Descriptive statistics were presented as mean ± standard deviation (SD), median (minimum–maximum), and number (percentage).

Within-group temporal comparisons were performed using the paired-samples *t* test for normally distributed data and the Wilcoxon signed-rank test for nonnormally distributed data. Comparisons among the 3 independent activity groups were performed using one-way analysis of variance (ANOVA) for normally distributed data and the Kruskal–Wallis test for nonnormally distributed data. When one-way ANOVA indicated a significant difference, Tukey post hoc tests were applied. To evaluate activity-related changes in each biomarker level, linear mixed-effects models adjusted for baseline biomarker levels were used. Categorical variables were compared using the Pearson χ^2^ test or the Fisher–Freeman–Halton exact test, as appropriate.

Relationships between continuous variables were evaluated using Pearson or Spearman correlation analyses, as appropriate. All correlation analyses were performed using baseline values. Statistical significance was defined as *p* < 0.05. An a priori power analysis using G*Power version 3.1.9.7 (Heinrich Heine University Düsseldorf, Düsseldorf, Germany) indicated that 40 patients per activity group were required to achieve 90% power for a two-sided paired-samples *t* test, assuming an effect size of d_z = 0.53 and α = 0.05.

### 2.5. Use of Generative Artificial Intelligence Tools

After all study procedures and scientific analyses had been completed, ChatGPT (GPT-5.6 Sol; OpenAI, San Francisco, CA, USA) was used for language and text editing and, under detailed instructions provided by the authors, to assist in the creation and refinement of the graphical abstract. The authors determined the scientific content, text, and visual elements to be included. The tool was not used for study design, data collection, statistical analysis, or interpretation of the results. All AI-assisted outputs were critically reviewed, edited, and approved by the authors.

## 3. Results

### 3.1. Study Population and Clinical Characteristics

A total of 120 patients were included in the final analysis, with 40 patients in each of the 1.11-GBq (Group 1), 3.70-GBq (Group 2), and 5.55-GBq (Group 3) activity groups. Baseline demographic characteristics are summarized in Table 1. Age did not differ significantly among the groups, whereas sex distribution differed significantly. Overall, 104 patients (86.7%) had papillary thyroid carcinoma, 14 (11.7%) had follicular thyroid carcinoma, and 2 (1.7%) had poorly differentiated thyroid carcinoma. Histologic subtype distribution did not differ significantly among the activity groups (*p* = 0.160).

### 3.2. Early Changes in Serum Renal Function Parameters

A statistically significant decrease in sCr levels and a corresponding increase in eGFR were observed on Day 7 compared with baseline in all three activity groups. In contrast, BUN levels remained unchanged in Group 1 but decreased significantly in Groups 2 and 3 (Table 2).

### 3.3. Urinary Parameters

uCr levels decreased significantly after treatment in all three groups. Absolute uAlb, uP, and uPro levels showed no consistent changes across the activity groups. Among the creatinine-normalized urinary parameters, only the uP/uCr ratio increased significantly in all groups, whereas the uAlb/uCr and uPro/uCr ratios remained unchanged (Table 3).

### 3.4. Dynamics of Novel Urinary Biomarkers Across RAI Activity Groups

All novel urinary biomarkers increased significantly from baseline to Day 1 in all RAI activity groups. For visual clarity, Figure 2 shows the individual dot plots for the 1.11-GBq group only. Biomarker levels partially declined by Day 7 and remained numerically above baseline; however, neither the Day 7 versus Day 1 nor the Day 7 versus baseline comparisons reached statistical significance. In contrast, all four uCr-normalized biomarker ratios remained significantly higher than baseline on Day 7. Absolute biomarker levels at baseline, Day 1, and Day 7 are presented in Table 4.

Day 1 percentage increases were numerically greater at higher activities (Figure 3), whereas absolute biomarker levels did not differ significantly among the activity groups (all *p* > 0.05; Table S1). However, after adjustment for baseline levels, no significant overall activity effect, time effect, or activity × time interaction was observed for KIM-1, netrin-1, cystatin C, or β2-microglobulin concentrations (Table 5). These findings indicate that the baseline-adjusted analysis did not support an independent activity-related effect on absolute biomarker concentrations.

### 3.5. Correlations Between Urinary Biomarkers and Renal Function Parameters

At baseline, KIM-1 and Netrin-1 showed strong negative correlations with eGFR and strong positive correlations with uCr, uAlb, uPro, sCr, and BUN. CysC and β2M demonstrated correlations in the same direction but of moderate strength. All correlations were statistically significant (all *p* < 0.001; Figure 4).

### 3.6. Thyroid Function Tests and Renal Function

TSH levels did not change significantly on Day 7 compared with baseline, whereas T3 and T4 levels increased significantly (both *p* < 0.05). At baseline, TSH was positively correlated with sCr (r = 0.684) and uCr (r = 0.632) and negatively correlated with eGFR (r = −0.521). T3 was positively correlated with eGFR (r = 0.510) and negatively correlated with sCr (r = −0.528). All reported correlations were statistically significant (all *p* < 0.001).

### 3.7. Hepatic and Hematologic Parameters

Liver-related biochemical parameters showed no consistent pattern of change across the activity groups. Lymphocyte and erythrocyte counts and hemoglobin levels decreased significantly in all RAI activity groups. Leukocyte counts decreased significantly in Groups 2 and 3 but remained unchanged in Group 1. Platelet counts decreased significantly in Groups 1 and 2 but remained unchanged in Group 3. Neutrophil counts remained unchanged in all groups (Table S2).

## 4. Discussion

This study was designed to investigate the early renal effects of RAI in humans, considering the RAI-related tubular injury and histopathological renal alterations reported in animal models [4,5,6]. The principal finding was a concordant increase in all four urinary tubular biomarkers on Day 1 across all administered-activity groups, despite the absence of deterioration in conventional renal function parameters. Percentage increases were numerically greater at higher administered activities and followed a consistent stepwise pattern across all four biomarkers. However, baseline-adjusted linear mixed-effects models showed no significant overall effect of activity group or activity group × time interaction. These findings provide prospective human evidence of an early subclinical tubular response that is not captured by conventional renal indices.

Biomarkers such as KIM-1, Netrin-1, CysC, and β2M have been shown in experimental and clinical studies to detect renal tubular stress earlier and with greater sensitivity than conventional renal function parameters such as sCr and BUN [9,10,11,12,13,14]. In the present study, the concordant Day 1 increases in all four biomarkers may reflect acute radiation-related stress in the tubular epithelium during the renal excretion of ^131^I [3,4]. The observation of changes in the same direction across biomarkers with different biologic characteristics and across all administered-activity groups strengthens the internal consistency of this finding. Other experimental studies reporting early and later cellular and histopathological alterations in renal tissue after RAI further support the biological plausibility of this interpretation [4,5,6]. The magnitude of these increases was considerably lower than the elevations reported in previous renal injury studies, and absolute biomarker levels showed a partial numerical decline by Day 7. Together with preserved conventional renal function, this comparatively modest response supports the interpretation of a limited subclinical tubular effect rather than overt renal injury. However, because the creatinine-normalized ratios remained significantly above baseline on Day 7 and follow-up was limited to 7 days, neither complete resolution nor the longer-term clinical significance of this response can be determined.

It is well recognized that creatinine normalization has limitations in the evaluation of urinary biomarkers, particularly when uCr excretion varies because of changes in hydration status or renal hemodynamics [16]. In the present study, uCr decreased after treatment, plausibly reflecting recommended post-treatment hydration together with altered renal hemodynamics. Nevertheless, the absolute concentrations of all four biomarkers increased significantly on Day 1 despite post-treatment hydration, which would be expected to dilute rather than increase urinary analyte concentrations [16]. This finding suggests that the early tubular biomarker response emerged despite the potential dilutional effect of hydration and that the actual response may have been more pronounced than measured. In addition, creatinine-normalized biomarker ratios remained significantly higher than baseline on Day 7. However, the decrease in uCr may have partially contributed to the magnitude of the normalized ratios [16]. Therefore, creatinine-normalized ratios should be interpreted cautiously.

No deterioration in conventional renal function parameters was observed after RAI therapy. sCr decreased and creatinine-derived eGFR increased on Day 7, whereas BUN decreased in the two higher-activity groups. Thyroid hormones have well-established effects on renal hemodynamics; in particular, correction of hypothyroidism may increase renal blood flow and glomerular filtration, with a corresponding decrease in serum creatinine levels [17]. Accordingly, the observed changes are considered more likely to reflect the combined effects of hormonal and hemodynamic changes following the resumption of levothyroxine therapy and post-treatment hydration than a true improvement in intrinsic renal function. The observed correlations of TSH with sCr and of T3 with eGFR are compatible with this hemodynamic mechanism, whereas reductions in BUN in the two higher-activity groups and in uCr across all groups suggest an additional contribution from hydration. Thus, conventional renal indices in this setting appear to be influenced by hormonal, hemodynamic, and dilutional factors and may not adequately reflect early renal effects at the cellular level.

Human data on the renal effects of RAI are limited, and available studies have largely focused on conventional renal function parameters [7,8]. Bhatkar et al. found no significant increase in microalbuminuria after RAI [7], whereas Yin et al., during a 5-year follow-up, reported no significant deterioration in mean sCr, BUN, or eGFR among patients with normal renal function at baseline, while a less favorable course was observed in patients with impaired renal function at baseline [8]. These findings are consistent with the absence of early deterioration in conventional renal function parameters in our study; however, they do not exclude subclinical changes that may be detectable using more sensitive tubular biomarkers.

In contrast to the tubular biomarker findings, uAlb and uPro showed no consistent increase across the administered-activity groups. The absence of a parallel increase in conventional urinary protein excretion, together with the concordant elevation of tubular biomarkers, supports a predominantly tubular response rather than overt glomerular injury. No significant changes were observed in liver function parameters. The hematologic changes showed trends consistent with previously reported effects of RAI therapy [18,19]; however, their magnitude was limited and unlikely to have been sufficient to affect renal parameters. These changes were therefore unlikely to account for the concordant increases observed in all four urinary tubular biomarkers.

This study has several important strengths. Its prospective design, balanced enrollment across the three administered-activity groups, an a priori sample size calculation supporting adequate statistical power, repeated within-patient sampling, assessment of the renal response using a multimarker panel, and analysis of all biomarker samples within the same assay run strengthen the methodologic rigor of the study. The originality of evaluating these biomarkers after RAI therapy further enhances its scientific relevance.

Several limitations should also be acknowledged. The single-center design and the absence of a control group consisting of thyroidectomized patients with DTC who did not receive RAI limit definitive attribution of the observed changes exclusively to RAI exposure. Radiation-safety constraints precluded Day 1 serum measurements, whereas limited urine volume prevented the assessment of some conventional urinary parameters on Day 1. Levothyroxine resumption and interindividual differences in hydration status may have influenced conventional renal parameters. Another important limitation is that serum CysC levels may be influenced by thyroid functional status [17,20]. Previous studies have reported that serum CysC levels may decrease in hypothyroidism and increase after initiation of levothyroxine therapy [17,20]. However, no human data are available regarding the direct effect of levothyroxine on urinary CysC. Nevertheless, changes in serum CysC levels may conceivably influence urinary CysC levels through related physiological mechanisms. In our study, baseline and Day 1 urine samples were obtained before levothyroxine was restarted; therefore, such an effect would not be expected to account for the increase in urinary CysC observed on Day 1. However, this potential hormonal effect should be considered a possible confounder when interpreting the Day 7 findings. Finally, the short follow-up period prevented evaluation of the longer-term clinical significance and reversibility of the observed tubular response.

The concordant increases observed across all four biomarkers support the interpretation of an early tubular response. Although percentage increases were numerically greater at higher administered activities, baseline-adjusted mixed-effects analyses did not support an independent activity–response relationship. In addition, the relatively limited magnitude of the biomarker changes and the absence of concomitant deterioration in conventional renal function indicate that these findings should not be interpreted as evidence of clinically meaningful renal injury. Although the present results do not warrant any change in routine RAI practice, they indicate that this early biomarker response should be validated in the general patient population using longer follow-up periods and more comprehensive study designs and should be further evaluated particularly in patients receiving repeated or high cumulative RAI activities or in those with reduced renal reserve.

## 5. Conclusions

In patients with DTC, concordant changes in urinary tubular biomarkers observed after RAI therapy, in the absence of deterioration in conventional renal function parameters, support the presence of an early subclinical tubular response. However, given the relatively modest magnitude of the biomarker changes and the lack of evidence for an independent activity–response relationship across administered-activity groups, these findings should not be interpreted as evidence of clinically significant renal injury.

Novel urinary biomarkers may represent a potential tool for evaluating early tubular changes after RAI that are not reflected by conventional renal function tests. However, the current findings do not support any change in routine RAI practice or renal monitoring strategies. The observed early tubular response should be confirmed in larger and longer-term studies and should be further evaluated particularly in patients with repeated or high cumulative RAI exposure or reduced renal reserve. Future studies in this field will help clarify the mechanisms underlying RAI-related renal effects and provide a scientific basis for investigating potential radioprotective strategies.

## Figures and Tables

**Figure 1:**
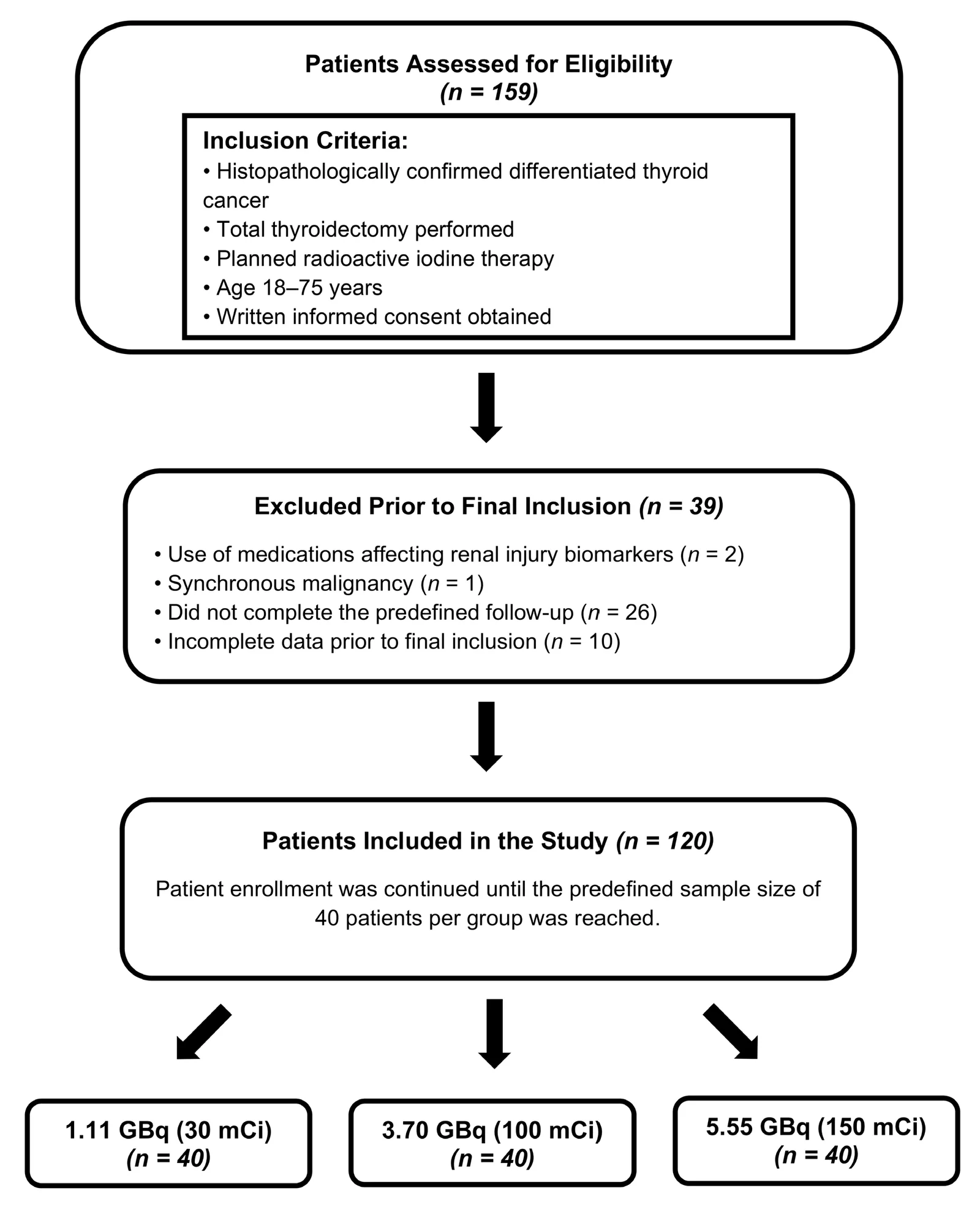
Flow diagram of patient recruitment, exclusion, and distribution by radioactive iodine activity.

**Figure 2:**
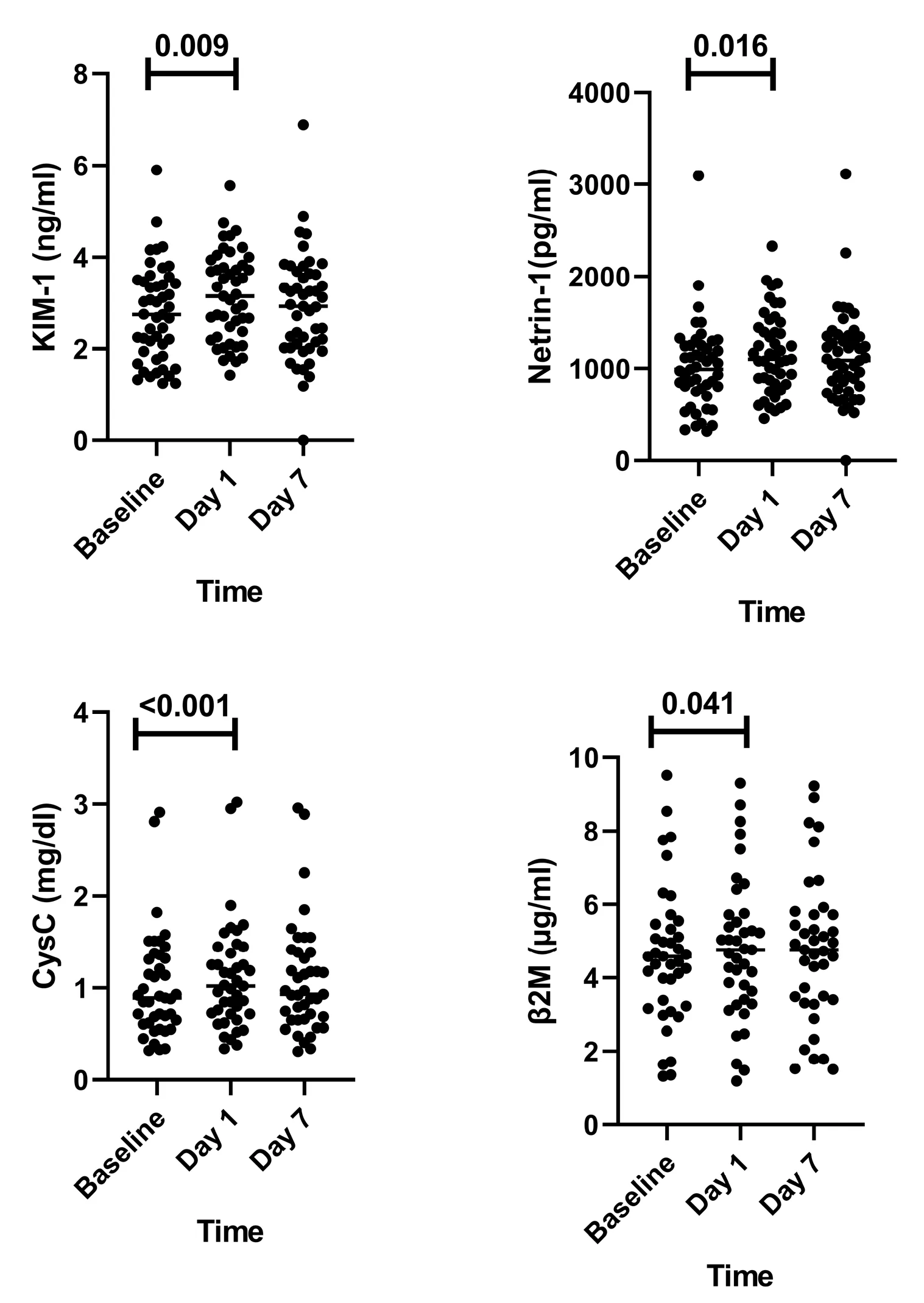
Individual values of urinary kidney injury molecule-1 (KIM-1), Netrin-1, cystatin C (CysC) and β2-microglobulin (β2M) at baseline, Day 1, and Day 7 in Group 1 (1.11 GBq) following radioactive iodine therapy. All urinary biomarkers increased on Day 1 and decreased by Day 7 but remained above baseline levels. *p*-values above the plots were calculated using paired-samples *t* tests and indicate baseline versus Day 1 comparisons (*p* < 0.05).

**Figure 3:**
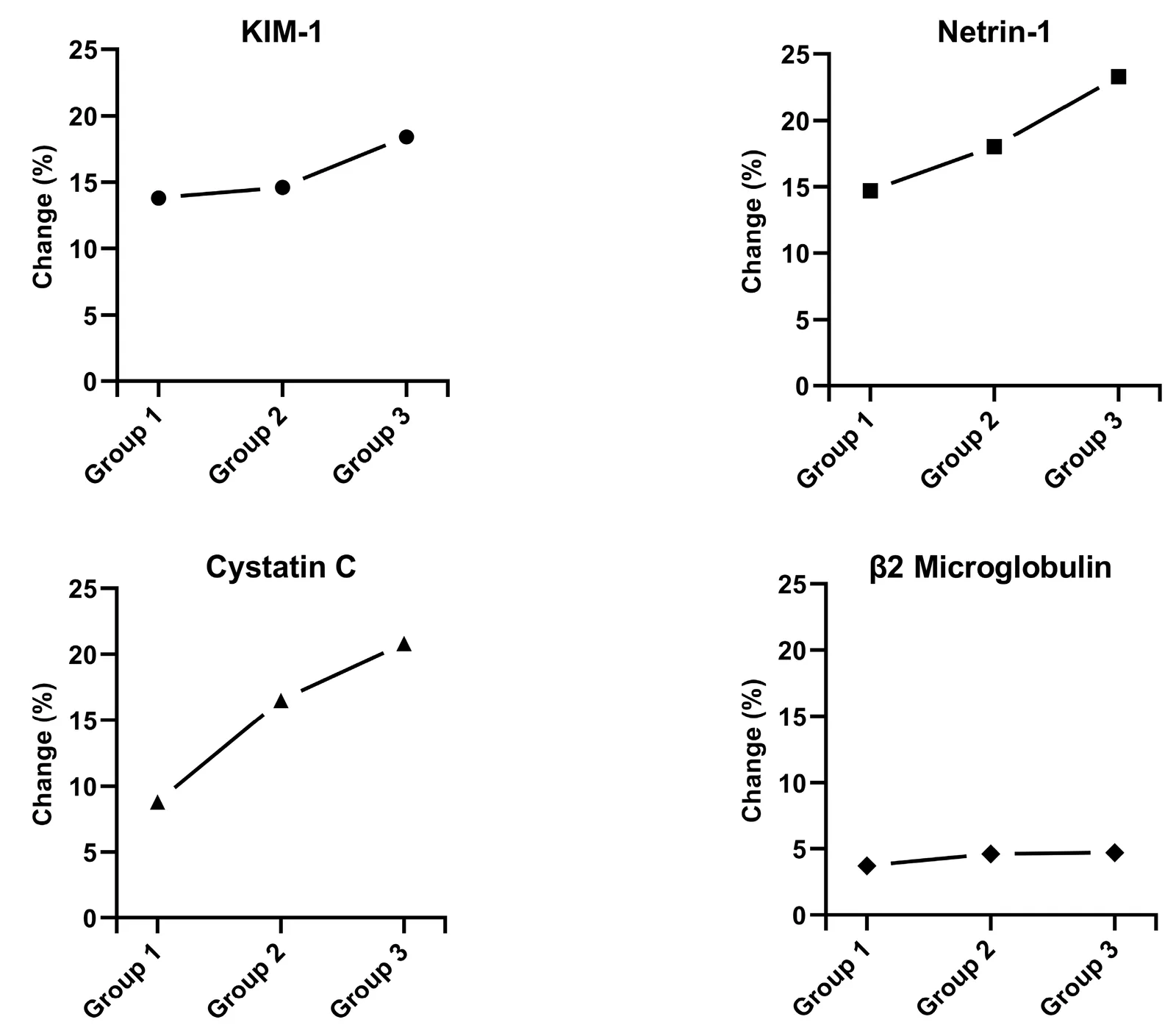
Percentage changes in urinary tubular injury biomarkers from baseline to Day 1 following radioactive iodine therapy. KIM-1 (●), Netrin-1 (■), CysC (▲), and β2-microglobulin (β2M; ◆). Group 1, 1.11 GBq; Group 2, 3.70 GBq; and Group 3, 5.55 GBq. Values are expressed as percentage change from baseline.

**Figure 4:**
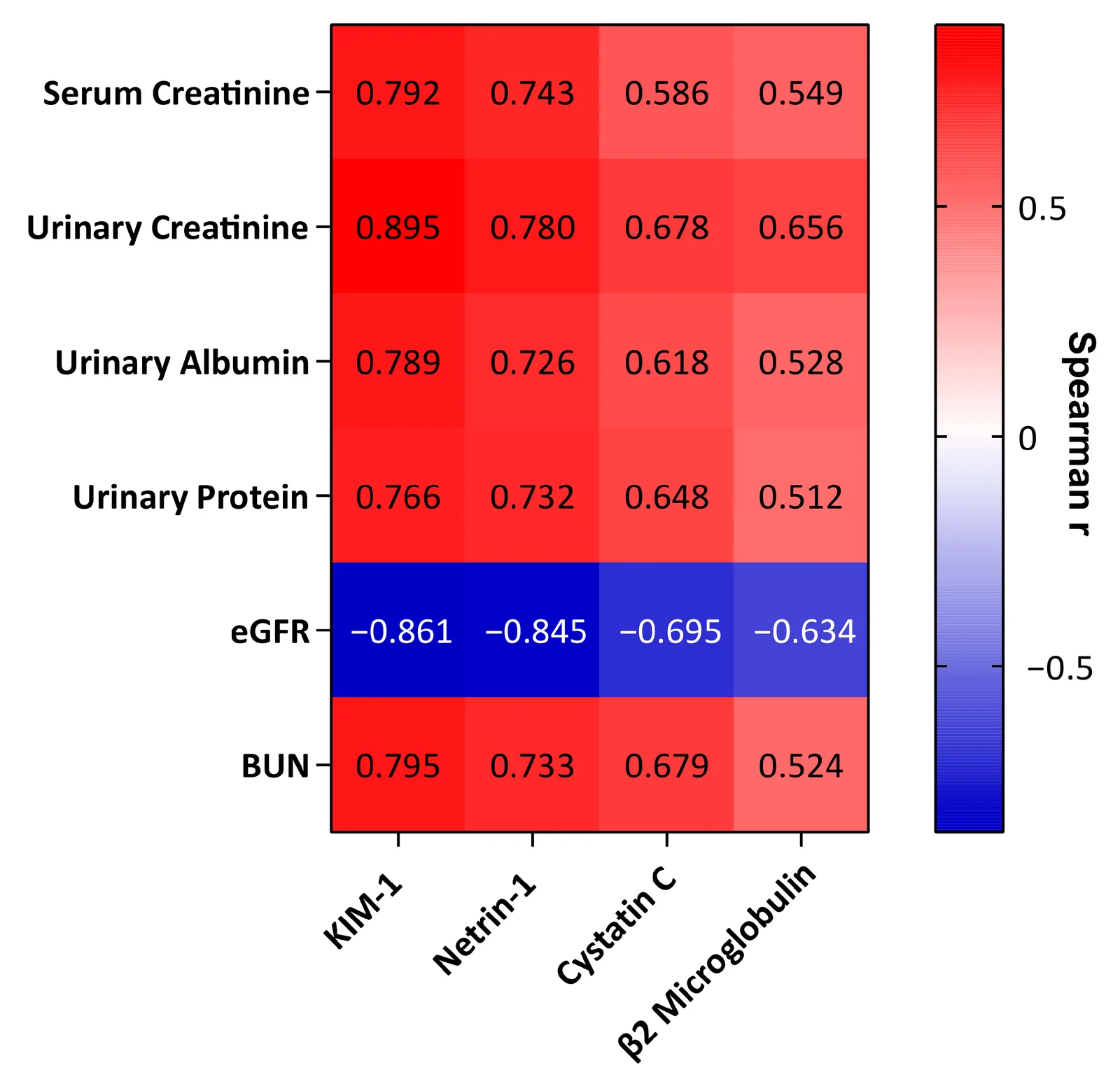
Heatmap showing Spearman correlations between urinary biomarkers and renal function parameters at baseline. Red and blue indicate positive and negative correlations, respectively. All correlations were statistically significant (*p* < 0.001). KIM-1 = kidney injury molecule-1; eGFR = estimated glomerular filtration rate; BUN = blood urea nitrogen.

**Table 1:** Patient Demographics.

Characteristic	Overall (*n* = 120)	1.11 GBq (*n* = 40)	3.70 GBq (*n* = 40)	5.55 GBq (*n* = 40)	*p*-Value
Age (y)	47.9 ± 14.2	48.2 ± 11.7	47.8 ± 14.4	47.8 ± 16.5	0.989
Sex, *n* (%)					0.025
Female	85 (70.8)	32 (80.0)	31 (77.5)	22 (55)	
Male	35 (29.2)	8 (20.0)	9 (22.5)	18 (45)	

Data are presented as mean ± SD. *p*-values were calculated using one-way analysis of variance for age and the Pearson χ^2^ test for sex. SD = standard deviation.

**Table 2:** Changes in Serum Renal Function Parameters at Baseline and Day 7 After Radioactive Iodine Therapy.

Parameter	Group 1 (*n* = 40, 1.11 GBq)		*p*	Group 2 (*n* = 40, 3.70 GBq)		*p*	Group 3 (*n* = 40, 5.55 GBq)		*p*
	Baseline	Day 7		Baseline	Day 7		Baseline	Day 7	
Creatinine (mg/dL)	0.91 ± 0.17	0.85 ± 0.16	0.002	0.97 ± 0.22	0.92 ± 0.19	0.024	1.06 ± 0.27	0.95 ± 0.23	0.001
eGFR (mL/min/1.73 m^2^)	83.9 ± 17.6	90.2 ± 17.6	0.001	81.9 ± 19.7	86.7 ± 19.5	0.015	78.3 ± 20.0	87.5 ± 20.8	<0.001
Blood Urea Nitrogen (mg/dL)	12.6 ± 3.6	12.8 ± 3.4	0.694	14.1 ± 4.6	12.7 ± 3.0	0.017	15.4 ± 5.8	13.6 ± 4.5	0.009

Data are presented as mean ± SD. *p*-values were calculated using paired-samples *t* tests and indicate within-group changes from baseline to Day 7. eGFR = estimated glomerular filtration rate. SD = standard deviation.

**Table 3:** Changes in Urinary Parameters From Baseline to Day 7 After Radioiodine Therapy.

Parameter	Group 1 (*n* = 40, 1.11 GBq)		*p*	Group 2 (*n* = 40, 3.70 GBq)		*p*	Group 3 (*n* = 40, 5.55 GBq)		*p*
	Baseline	Day 7		Baseline	Day 7		Baseline	Day 7	
uCr (mg/dL)	99.4 (10.3–363.7)	54.9 (14.9–458.9)	0.009 ^b^	129.0 ± 93.1	96.6 ± 61.7	0.026 ^a^	169.2 ± 117.3	107.0 ± 85.5	0.008 ^a^
uAlb (mg/L)	0.9 (0–177.1)	0.9 (0–13.3)	0.754 ^b^	0.8 (0–57.7)	0.7 (0–7.7)	0.554 ^b^	1.2 (0–50.3)	1.1 (0–13.0)	0.315 ^b^
uP (mg/dL)	37.1 ± 30.7	33.7 ± 29.0	0.555 ^b^	38.6 ± 32.9	38.4 ± 30.3	0.966 ^a^	46.5 ± 32.4	34.3 ± 20.6	0.052 ^a^
uPro (mg/dL)	6.5 (0.4–22.7)	4.4 (0.7–19.6)	0.012 ^b^	6.9 (1.3–143.2)	7.4 (0.8–271.3)	0.570 ^b^	8.6 (0.8–70.5)	6.7 (1.3–43)	0.315 ^b^
uP/uCr (mg/g Cr)	354.3 ± 188.9	490.7 ± 197.1	0.005 ^a^	302.6 ± 147.9	418.1 ±185.8	<0.001 ^a^	283.9 ± 117.0	371.0 ± 158.0	0.004 ^a^
uAlb/uCr (mg/g Cr)	5.9 (0.1–1142.1)	12.7 (0–557.5)	0.141 ^b^	8.1 (0–182.2)	6.2 (0–90.3)	0.975 ^b^	9.3 (0–109.4)	10.2 (0–182.7)	0.364 ^b^
uPro/uCr (mg/g Cr)	60.5 (28.0−368.2)	75.5 (20.1–197.3)	0.096 ^b^	74.9 (24.2−970.7)	82.1 (0−2250.5)	0.112 ^b^	63.5 (17.8−586.4)	67.0 (31.7–765.5)	0.103 ^b^

Data are presented as mean ± SD or median (range). *p*-values indicate within-group temporal comparisons between baseline and Day 7. ^a^ Paired-samples *t* test; ^b^ Wilcoxon signed-rank test. uCr = urinary creatinine; uAlb = urinary albumin; uP = urinary phosphate; uPro = urinary protein.

**Table 4:** Changes in Urinary Tubular Injury Biomarkers Following Radioactive Iodine Therapy.

Biomarker	Time Point	Group 1 (*n* = 40, 1.11 GBq)	Group 2 (*n* = 40, 3.70 GBq)	Group 3 (*n* = 40, 5.55 GBq)
KIM-1 (ng/mL)	Baseline	2.76 ± 1.04	2.95 ± 1.19	2.55 ± 1.03
	Day 1	3.14 ± 0.96	3.38 ± 1.07	3.02 ± 0.95
	Day 7	2.91 ± 1.16	3.29 ± 1.16	2.75 ± 1.03
	*p*-value *	0.009/0.428/0.093	0.024/0.086/0.657	0.037/0.215/0.237
Netrin-1 (pg/mL)	Baseline	1015.16 ± 478.49	976.27 ± 423.87	1135.08 ± 521.92
	Day 1	1164.45 ± 434.56	1151.89 ± 464.72	1399.46 ± 629.83
	Day 7	1118.62 ± 494.24	1079.19 ± 596.68	1254.36 ± 468.32
	*p*-value *	0.016/0.084/0.413	0.021/0.279/0.444	0.049/0.079/0.109
Cystatin C (mg/dL)	Baseline	1.02 ± 0.57	0.91 ± 0.48	0.96 ± 0.47
	Day 1	1.11 ± 0.58	1.06 ± 0.55	1.16 ± 0.64
	Day 7	1.09 ± 0.60	1.02 ± 0.51	1.04 ± 0.58
	*p*-value *	<0.001/0.072/0.544	0.01/0.091/0.498	0.004/0.255/0.094
β2-Microglobulin (µg/mL)	Baseline	4.62 ± 1.88	4.39 ± 1.98	4.23 ± 2.32
	Day 1	4.79 ± 1.92	4.59 ± 2.14	4.43 ± 2.21
	Day 7	4.77 ± 1.98	4.46 ± 1.97	4.36 ± 2.24
	*p*-value *	0.041/0.109/0.716	0.016/0.155/0.120	0.018/0.086/0.107

Values are presented as mean ± SD. * *p*-values represent comparisons between baseline vs. day 1/baseline vs. day 7/day 1 vs. day 7, respectively (paired-samples *t* test). KIM-1 = kidney injury molecule-1, SD = standard deviation.

**Table 5:** Baseline-Adjusted Linear Mixed-Effects Model Analysis of Urinary Biomarker Levels According to Activity Group.

Biomarker	Activity Group	Baseline Mean ± SD	Day 1 Adjusted Mean (95% CI)	Day 7 Adjusted Mean (95% CI)	Activity Group Effect *p*	Time Effect *p*	Activity Group × Time *p*	Log-Transformed Sensitivity Analysis *p* *
Kidney injury molecule-1 (KIM-1) (ng/mL)	Group 1 (1.11 GBq)	2.76 ± 1.04	3.14 (2.87–3.42)	2.98 (2.70–3.25)	0.502	0.375	0.804	0.434/0.180/0.641
	Group 2 (3.70 GBq)	2.95 ± 1.19	3.29 (3.00–3.58)	3.20 (2.91–3.49)				
	Group 3 (5.55 GBq)	2.55 ± 1.03	3.11 (2.81–3.41)	2.84 (2.54–3.14)				
Netrin-1 (pg/mL)	Group 1 (1.11 GBq)	1015.2 ± 478.5	1180.1 (1056.7–1303.5)	1154.0 (1029.4–1278.7)	0.392	0.237	0.546	0.458/0.265/0.661
	Group 2 (3.70 GBq)	976.3 ± 423.9	1190.3 (1054.6–1326.0)	1117.6 (981.9–1253.3)				
	Group 3 (5.55 GBq)	1135.1 ± 521.9	1345.0 (1212.4–1477.7)	1199.9 (1067.3–1332.6)				
Cystatin C (mg/dL)	Group 1 (1.11 GBq)	1.02 ± 0.57	1.06 (0.96–1.17)	1.04 (0.93–1.15)	0.744	0.248	0.561	0.785/0.139/0.505
	Group 2 (3.70 GBq)	0.91 ± 0.48	1.11 (1.00–1.22)	1.06 (0.95–1.17)				
	Group 3 (5.55 GBq)	0.96 ± 0.47	1.16 (1.06–1.26)	1.05 (0.95–1.15)				
β2-microglobulin (µg/mL)	Group 1 (1.11 GBq)	4.62 ± 1.88	4.59 (4.43–4.76)	4.58 (4.41–4.74)	0.780	0.114	0.440	0.936/0.365/0.884
	Group 2 (3.70 GBq)	4.39 ± 1.98	4.62 (4.47–4.76)	4.48 (4.34–4.63)				
	Group 3 (5.55 GBq)	4.23 ± 2.32	4.60 (4.45–4.76)	4.53 (4.38–4.69)				

Baseline values are presented as mean ± SD; Day 1 and Day 7 values are baseline-adjusted estimated marginal means (95% CI). Linear mixed-effects models included baseline biomarker level, activity group, time, and the activity group × time interaction, with a subject-specific random intercept; overall effects were evaluated using likelihood-ratio tests. Each activity group included 40 patients. * Log-transformed sensitivity analysis *p*-values are reported in the following order: activity-group effect/time effect/activity group × time interaction. CI, confidence interval; SD, standard deviation.

## Data Availability

Data are available on request due to privacy and ethical restrictions. The data supporting the findings of this study are available from the corresponding author upon reasonable request, subject to institutional and ethical approval.

## Supplementary Materials

The following supporting information can be downloaded at: https://www.mdpi.com/article/10.3390/diagnostics16193123/s1, Table S1: Comparison of urinary KIM-1, netrin-1, cystatin C, and beta-2-microglobulin levels among radioactive iodine activity groups; Table S2: Changes in liver-related biochemical and hematological parameters from baseline to Day 7 after radioactive iodine therapy, stratified by administered activity group.
