# Supplementary material for: Early Renal Effects After Radioactive Iodine Therapy in Differentiated Thyroid Cancer: A Prospective Observational Study
Source: Diagnostics (Basel). 2026 Sep 25;16(19):3123. doi: 10.3390/diagnostics16193123 (PMC13664341; doi:10.3390/diagnostics16193123)
Supplement: Supplementary file 1 [file diagnostics-16-03123-s001.zip › diagnostics-4520339-supplementary.pdf]

Supplementary Materials

Table S1. Comparison of urinary KIM-1, netrin-1, cystatin C, and beta-2-microglobulin levels among radioactive iodine activity groups

Biomarker	Time point	Group 1 1.11 GBq (*n* = 40)	Group 2 3.70 GBq (*n* = 40)	Group 3 5.55 GBq (*n* = 40)	*p* value
Kidney injury molecule-1 (ng/mL)	Baseline	2.74 (1.25-5.90)	2.95 (1.26-6.85)	2.33 (1.14-5.79)	0.297[a]
	Day 1	3.14 ± 0.96	3.38 ± 1.07	3.02 ± 0.95	0.144[b]
	Day 7	2.93 (1.43-6.89)	3.21 (1.38-7.53)	2.61 (1.15-7.09)	0.061[a]
	Baseline–Day 1 increase (%)	13.8	14.6	18.4	-
Netrin-1 (pg/mL)	Baseline	992.64 (314.24-3093.84)	877.34 (308.93-1902.40)	1056.84 (288.03-3466.59)	0.258[a]
	Day 1	1103.20 (455.63-2333.27)	1124.13 (311.05-2723.05)	1346.08 (333.63-3673.84)	0.080[a]
	Day 7	1087.61 (378.27-3115.70)	942.36 (362.41-3248.34)	1242.15 (433.38-2415.36)	0.060[a]
	Baseline–Day 1 increase (%)	14.7	18.0	23.3	-
Cystatin C (mg/dL)	Baseline	0.89 (0.32-2.91)	0.92 (0.20-1.85)	0.96 (0.23-2.02)	0.832[a]
	Day 1	1.02 (0.34-3.02)	0.98 (0.29-2.74)	1.11 (0.34-3.15)	0.902[a]
	Day 7	0.93 (0.31-2.96)	0.97 (0.25-2.22)	1.03 (0.19-2.94)	0.976[a]
	Baseline–Day 1 increase (%)	8.8	16.5	20.8	-
Beta-2-microglobulin (μg/mL)	Baseline	4.58 (1.32-9.52)	4.51 (0.99-8.44)	3.57 (1.03-9.10)	0.601[a]
	Day 1	4.79 ± 1.92	4.59 ± 2.14	4.43 ± 2.21	0.744[b]
	Day 7	4.77 ± 1.98	4.46 ± 1.97	4.36 ± 2.24	0.648[b]
	Baseline–Day 1 increase (%)	3.7	4.6	4.7	-

Data are presented as mean ± standard deviation or median (minimum-maximum), as appropriate. *p* values compare the three activity groups at the corresponding time point. a Kruskal-Wallis test. b One-way analysis of variance with Tukey's post hoc test. Percentage increase refers to the change from baseline to Day 1. KIM-1, kidney injury molecule-1; CysC, cystatin C; β2M, beta-2-microglobulin.

Table S2. Changes in liver-related biochemical and hematological parameters from baseline to Day 7 after radioactive iodine therapy, stratified by administered activity group

Variable	Time point	Group 1 1.11 GBq (*n* = 40)	Group 2 3.70 GBq (*n* = 40)	Group 3 5.55 GBq (*n* = 40)
A. Liver-related biochemical parameters				
Aspartate aminotransferase (U/L)	Baseline	27.65 ± 10.34	29.36 ± 10.11	27 (15-80)
	Day 7	32.43 ± 17.11	30.20 ± 12.21	26.30 (12-87)
	p value	0.002[a]	0.453[a]	0.446[b]
Alanine aminotransferase (U/L)	Baseline	20.80 ± 6.99	26.73 ± 16.17	30.46 ± 19.86
	Day 7	25.66 ± 11.03	29.26 ± 22.89	34.42 ± 21.57
	p value	<0.001[a]	0.224[a]	0.023[a]

Lactate dehydrogenase (U/L)	Baseline	236.66 ± 37.38	247.33 ± 52.78	247.64 ± 90.81
	Day 7	249.34 ± 48.89	246.44 ± 55.51	244.36 ± 82.47
	p value	0.014[a]	0.868[a]	0.518[a]
Total protein (g/dL)	Baseline	7.38 ± 0.41	7.51 ± 0.39	7.53 ± 0.40
	Day 7	7.35 ± 0.42	7.46 ± 0.36	7.40 ± 0.46
	p value	0.578[a]	0.440[a]	0.052[a]
Prealbumin (g/L)	Baseline	0.29 ± 0.05	0.30 ± 0.04	0.30 ± 0.05
	Day 7	0.31 ± 0.04	0.30 ± 0.05	0.29 ± 0.07
	p value	0.016[a]	0.990[a]	0.538[a]
Albumin (g/dL)	Baseline	4.46 ± 0.25	4.49 ± 0.31	4.56 ± 0.29
	Day 7	4.45 ± 0.24	4.43 ± 0.23	4.47 ± 0.37
	p value	0.825[a]	0.247[a]	0.022[a]
B. Hematological parameters				
	Baseline	7.23 ± 1.74	7.78 ± 1.88	7.54 ± 2.13
Leukocyte count ($\times 10^9$/L)	Day 7	7.03 ± 1.69	6.87 ± 1.44	6.62 ± 1.97
	p value	0.309[a]	0.001[a]	0.001[a]
	Baseline	2.60 ± 0.76	2.65 ± 0.74	2.73 ± 0.83
Lymphocyte count ($\times 10^9$/L)	Day 7	2.29 ± 0.69	1.91 ± 0.49	1.55 ± 0.55
	p value	0.001[a]	<0.001[a]	<0.001[a]
	Baseline	4.00 ± 1.03	4.53 ± 1.64	4.14 ± 1.62
Neutrophil count ($\times 10^9$/L)	Day 7	4.12 ± 1.09	4.38 ± 1.02	4.39 ± 1.75
	p value	0.352[a]	0.978[b]	0.335[a]
	Baseline	4.95 ± 0.51	5.14 ± 0.43	5.16 ± 0.61
Erythrocyte count ($\times 10^{12}$/L)	Day 7	4.78 ± 0.48	4.93 ± 0.46	4.89 ± 0.54
	p value	<0.001[a]	<0.001[a]	<0.001[a]
	Baseline	13.93 ± 1.53	14.63 ± 1.67	14.89 ± 2.13
Hemoglobin (g/dL)	Day 7	13.56 ± 1.52	14.17 ± 1.78	14.12 ± 1.95
	p value	<0.001[a]	<0.001[a]	<0.001[a]
	Baseline	276.07 ± 55.18	283.30 ± 72.93	268.05 ± 70.16
Platelet count ($\times 10^9$/L)	Day 7	264.05 ± 56.28	262.63 ± 69.01	256.46 ± 54.25
	p value	0.021[a]	<0.001[a]	0.146[a]

Data are presented as mean ± standard deviation or median (minimum-maximum), as appropriate. *p* values compare baseline and Day 7 within each activity group. a Paired-samples t-test. b Wilcoxon signed-rank test.
